# Effectiveness of Perfusion Index ratio and End-Diastolic Velocity ratio in evaluating the efficacy of Interscalene Brachial Plexus Block: a prospective observational study

**DOI:** 10.3389/fmed.2025.1571171

**Published:** 2025-04-03

**Authors:** Li Hu, Jintao Sun, Bin Zhang, Xiaoyan Ye, Jian Lu, Zhipeng Zhu, Hongmei Zhou

**Affiliations:** ^1^Department of Anesthesiology, The Second Affiliated Hospital of Jiaxing University, Jiaxing, China; ^2^Key Laboratory of Basic Research and Clinical Transformation of Perioperative Precision Anesthesia, Jiaxing, China; ^3^Medical College, Jiaxing University, Jiaxing, China

**Keywords:** nerve block efficacy, regional anesthesia, Perfusion Index ratio, End-Diastolic Velocity ratio, Interscalene Brachial Plexus Block, receiver operating characteristic curve

## Abstract

**Objective:**

There is a lack of reliable indicators for evaluating the success of ultrasound-guided Interscalene Brachial Plexus Block (ISBPB). This study investigates the effectiveness of Perfusion Index (PI) ratio and End-Diastolic Velocity (EDV) ratio for early assessment of ISBPB effects.

**Methods:**

Eighty-nine patients, aged 18–65 with BMI 18–24 kg/m^2^ and ASA grade I or II, underwent elective unilateral shoulder arthroscopic surgery. They received ultrasound-guided ISBPB with 15 mL local anesthetic (10 mL ropivacaine, 5 mL lidocaine). Patients were categorized into successful and failure groups based on needle test results after 30 min. PI and EDV of the brachial artery were recorded at baseline and at 5, 10, 15, 20, 25, and 30 min post-block. PI and EDV ratios were calculated by dividing values at each time by baseline. ROC curves were plotted at 5 and 10 min, and AUROC with 95% CI was calculated to assess block efficacy.

**Results:**

Of 89 patients, 3 were excluded due to data loss and 2 withdrew, leaving 84 patients. Of these, 70 (83.3%) had successful blocks. In the successful group, both PI and EDV ratios on the blocked side significantly increased 5 min after the procedure. The PI ratio at 5 min had an AUROC of 0.894 (95% CI: 0.816–0.972), with a threshold of 1.22, sensitivity of 84.3%, and specificity of 85.7%. The EDV ratio had an AUROC of 0.706 (95% CI: 0.553–0.860), with a threshold of 1.32, sensitivity of 92.9%, and specificity of 50%. At 10 min, the PI ratio for assessing ISBPB impact had an AUROC of 0.901 (95% CI: 0.828–0.974), with a threshold of 1.4, sensitivity of 74.3%, and specificity of 92.9%. The AUROC for the EDV ratio was 0.799 (95% CI: 0.6788–0.921) with a threshold of 1.54, sensitivity of 92.9%, and specificity of 57.1%. The PI ratio at 5 min had a significantly higher AUROC than the EDV ratio, but no significant difference was found between PI ratios at 5 and 10 min.

**Conclusion:**

Both PI ratio and EDV ratio assess ISBPB efficacy. The PI ratio provides a more precise evaluation, with optimal assessment at 5 min post-procedure.

**Clinical trial registration:**

Chinese Clinical Trial Registry: ChiCTR2200066874.

## 1 Introduction

Regional nerve block (RNB) techniques are essential in perioperative multimodal analgesia, offering pain relief, reduced opioid use, and improved patient satisfaction (1, 2). However, clinical practice lacks early, accurate, and objective evaluation indicators. Instead, it often relies on subjective assessments of pain, temperature, and motor changes, which can be imprecise and delayed (3). Since sympathetic blockade precedes sensory and motor effects, objective measures like the PI and End-Diastolic Velocity (EDV) have gained interest (4, 5). PI, derived from pulse oximetry, reflects tissue perfusion changes due to vasodilation after sympathetic blockade, while EDV, measured via Doppler ultrasound, tracks hemodynamic shifts (6). Adjusting these values to baseline ratios (PI ratio and EDV ratio) minimizes individual variability, enhancing assessment accuracy (7).

This study examines the dynamic changes in PI and EDV ratios following Interscalene Brachial Plexus Block (ISBPB), evaluates PI ratio as an early assessment tool, and compares their AUROC to provide anesthesiologists with a reliable method for improving clinical decision-making and patient outcomes.

## 2 Materials and methods

### 2.1 Study design

This prospective observational trial was conducted at the Second Affiliated Hospital of Jiaxing University between October and December 2023. This study was approved by the Ethics Committee of Second Affiliated Hospital of Jiaxing University (China, Approval no. JXEY-2023SW088). The study was registered in the Chinese Clinical Trial Registry.^1^ All participants in our study were obtained the informed consent.

### 2.2 Study population

This prospective observational trial was conducted on 89 patients who underwent unilateral arthroscopic shoulder surgery. The inclusion criteria were as follows: ① patients aged between 18 and 65, ② American Society of Anesthesiologists (ASA) I–II level, ③ body mass index (BMI) between 18 and 24 kg/m^2^. The exclusion criteria were as follows: ① preoperative use of vasoactive drugs and analgesics, ② peripheral vascular diseases, ③ paresthesia, ④ allergy to local anesthetics, ⑤ coagulation dysfunction, ⑥ presence of psychiatric symptoms.

### 2.3 Study intervention

All patients underwent an 8-h fasting period and were restricted from fluid intake for 2 h prior to the administration of anesthesia. The ambient temperature was consistently maintained between 24 and 26°C. Upon arrival in the anesthesia preparation area, peripheral venous access was established for each patient, and oxygen was administered via nasal catheter at a rate of 2 L/min. Standard monitoring protocols were implemented, including non-invasive blood pressure measurement, electrocardiography, and pulse oximetry. To mitigate emotional responses, midazolam was administered intravenously at a dosage of 0.03 mL·kg^−1^ for sedation.

### 2.4 Measurement of Perfusion Index

The probe of pulse oximetry (MX550, Philips, Amsterdam, Netherlands) for monitoring PI was placed on the index finger of the surgical side and wrapped by opaque black cloth to avoid light interference and reduce heat loss. on PI. The anesthesiologist waited 10 min for the baseline to stabilize and then the ISBPB was performed.

### 2.5 Measurement of EDV

**Figure 1:**
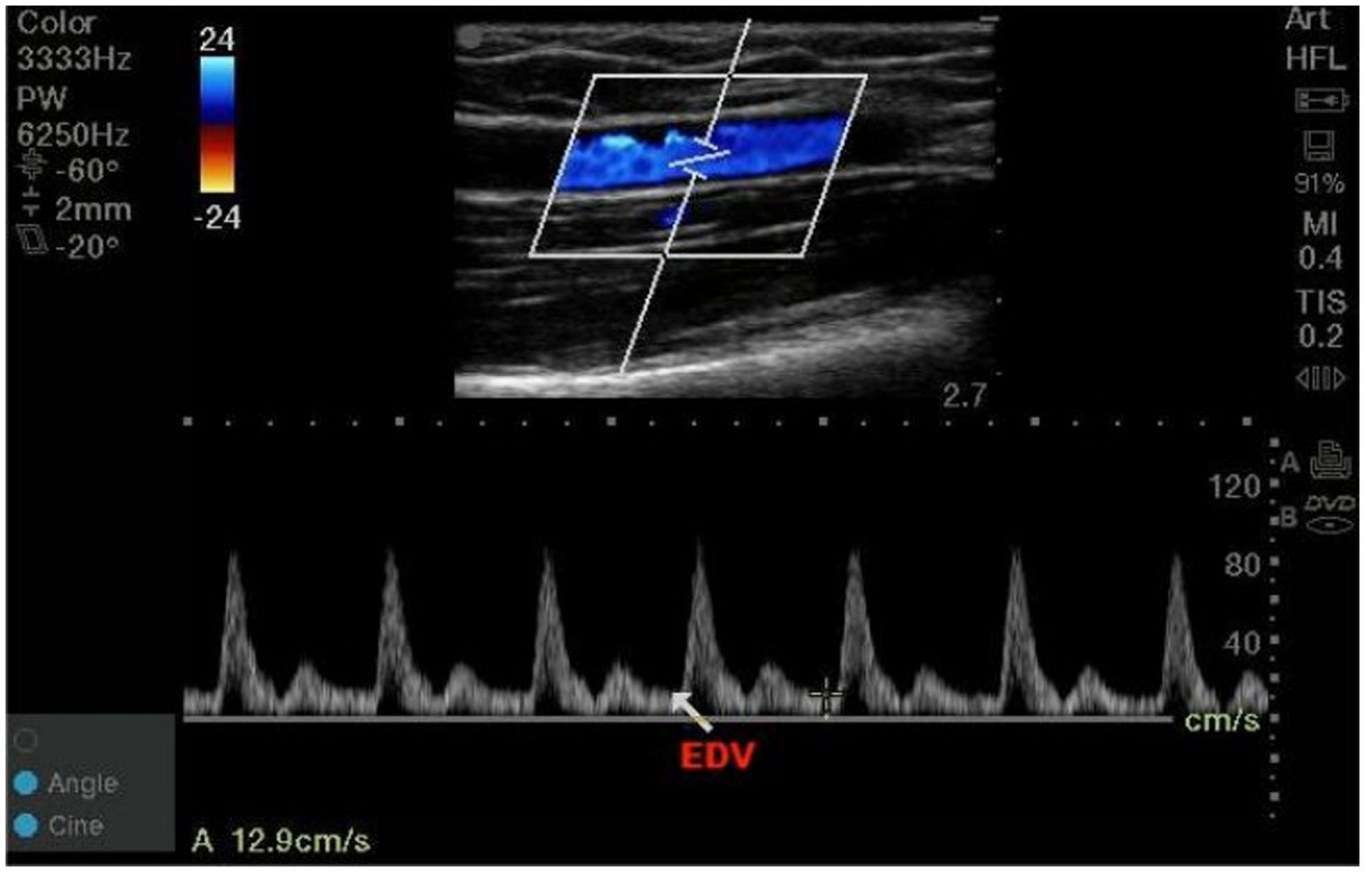
Diagram representation of the EDV measurements.

The high frequency linear-array transducer was placed 3 cm proximal to the antecubital fossa to target the brachial artery. A two-dimensional sagittal scan of the brachial artery was performed, followed by activation of the pulsed-wave Doppler mode. The angle correction cursor should be parallel to the direction of blood flow, and then the sample volume length was adjusted to 2 mm (between 1/3 and 1/2 of the inner diameter) at the center of vessels. The Doppler angle that between the ultrasound beam and the direction of blood flow was maintained less than or equal to 60°. The EDV was obtained by tracing three consecutive cardiac cycles and averaged. The high frequency linear-array transducer was returned to the same position by marking on the skin to measure in subsequent observation periods (Figure 1). All measurements were performed by the same anesthesiologist who systematically learned vascular ultrasound examination.

### 2.6 Ultrasound-guided ISBPB procedure

The patient is placed in a supine position with the head slightly turned to the opposite side. The operator stands at the head of the patient, and the portable ultrasound machine is placed on the non-blocked side. Following sterile principles, the area to be blocked is disinfected and draped. A high-frequency linear probe (6–13 MHz) is chosen, and ultrasound gel is evenly applied. A sterile sheath is fitted over the probe, and the depth is set to 3–4 cm. The probe is positioned in the supraclavicular fossa, parallel to the distal clavicle and perpendicular to the skin of the neck. The goal is to locate the medial, posterior, and lateral fascicles of the brachial plexus, as well as the pleura beneath them, by scanning from the proximal brachial plexus to the root.

Once the brachial plexus is visualized, the probe is moved upwards along the neck. When the brachial plexus is located between the anterior and middle scalene muscles, this is identified as the interscalene space. The probe is then stopped, and the insertion point is located about 1 cm lateral to the ultrasound probe. Before puncture, color Doppler is activated to check for the presence of any vessels along the needle path. The needle is advanced using the in-plane technique, slowly penetrating the skin from lateral to medial. The needle is clearly visible throughout the procedure in the ultrasound field, avoiding any vessels along the puncture path.

The single-use nerve block needle (Specifications: 22G 0.7*50 mm, Model: II, Production Batch: 202201019, Manufacturer: Henan TuoRen Medical Device Group Co., Ltd.) is carefully advanced through the middle scalene muscle until it reaches the anterior edge of the middle scalene muscle and the posterior edge of the brachial plexus bundle. Once the needle is positioned, aspiration is performed to ensure there is no blood or air. After confirming this, 7.5 mL of local anesthetic (a mixture of 5 mL Ropivacaine Hydrochloride [75 mg/10 mL, Batch: H20140764, Manufacturer: AstraZeneca AB, Sweden] and 2.5 mL Lidocaine Hydrochloride [0.1 g/5 mL, Batch: F220924C, Manufacturer: Hunan Kelun Pharmaceutical Co., Ltd.]) is injected under continuous real-time ultrasound monitoring. The spread of the local anesthetic is observed to ensure adequate diffusion.

The needle is then retracted to the subcutaneous level, and its angle adjusted to approach the brachial plexus from above, closely aligning with the target nerves. After confirming no blood or air on aspiration, the same concentration and volume of the Ropivacaine-Lidocaine mixture is injected, ensuring the local anesthetic envelops the target nerves and spreads adequately under real-time ultrasound monitoring. This procedure is performed by an experienced anesthesiologist proficient in ultrasound-guided regional nerve blocks.

### 2.7 Assessment of the block results

The pain sensation in the area innervated by the musculocutaneous nerve, radial nerve, median nerve, and ulnar nerve was assessed through needling tests using a No. 25 blunt needle. The corresponding areas of innervation for each nerve are as follows: ① Musculocutaneous nerve dermatome: the skin area along the lateral side of the forearm to the wrist; ② Radial nerve absolute dermatome: the area on the dorsum of the hand, particularly the first web space; ③ Median nerve absolute dermatome: the distal tips of the index and middle fingers; ④ Ulnar nerve absolute dermatome: the little finger. A 3-point scoring system was used: 2 = normal sensation; 1 = diminished sensation; 0 = absence of sensation. The lowest level of assessment for the above nerves was considered the blockade effect. Successful ultrasound-guided ISBPB was defined as the loss of sensation in the skin areas innervated by at least three target nerves 30 min after performing the block. Based on the ultrasound-guided ISBPB needle test results, the cases were categorized into successful group and failed group. The evaluation of the ISBPB effect was conducted and recorded by a senior anesthesiologist who did not perform the nerve block.

### 2.8 Outcomes

Collect and document the patient’s demographic and surgical information, including age, gender, height, weight, body mass index (BMI), American Society of Anesthesiologists (ASA) classification, and the surgical site (left or right). The ultrasound-guided ISBPB procedure will be conducted by an anesthesiologist with training in ultrasound vascular examination but without prior experience in nerve block procedures. Baseline measurements of EDV and PI will be recorded by the anesthesiologist 1 min prior to the ultrasound-guided ISBPB. Subsequent recordings of PI and EDV will be taken at intervals of 5, 10, 15, 20, 25, and 30 min following the procedure. The PI and EDV ratios will be calculated for the post-procedural observation period, defined as the PI or EDV value during the observation period divided by the baseline value recorded 1 min before the ISBPB. Measurements of PI and EDV, including the baseline, will be documented at 5-min intervals up to 30 min post-ISBPB.

### 2.9 Statistical analysis

The PASS 15.0 software was used to calculate the required sample size. The area under the ROC curve (AUC) was set at 0.8, with the null hypothesis set at 0.5. Referring to previous studies, the failure rate of the nerve block was reported as 10%. Results from a preliminary experiment showed a failure rate of 8%, consistent with previous literature. Therefore, the failure rate for this block was set at 8%. With a significance level of 0.05, statistical power of 0.8, and a 15% allowance for sample attrition, at least 89 cases (including at least 7 failures) were required.

The collected data were imported and processed using SPSS 23.0 statistical software. The normality of continuous variables was assessed using the Shapiro–Wilk test and P–P plots. Continuous data conforming to a normal distribution were expressed as mean ± standard deviation, and group comparisons were conducted using independent sample *t*-tests. For the PI ratio and EDV ratio at different time points after ultrasound-guided ISBPB, repeated-measures one-way ANOVA was applied. Categorical data were presented as frequencies and percentages (%) and analyzed using the *χ*^2^ test.

To evaluate the effectiveness of ultrasound-guided ISBPB, receiver operating characteristic (ROC) curves were plotted for the PI ratio and EDV ratio in response to the block’s efficacy. Sensitivity and specificity were calculated based on the point with the highest Youden index on the ROC curve, along with the diagnostic threshold, AUC, and 95% confidence interval (CI). The DeLong test was used to compare the predictive value of the AUCs for the PI and EDV ratios. A *p* value of <0.05 was considered statistically significant.

## 3 Results

**Figure 2:**
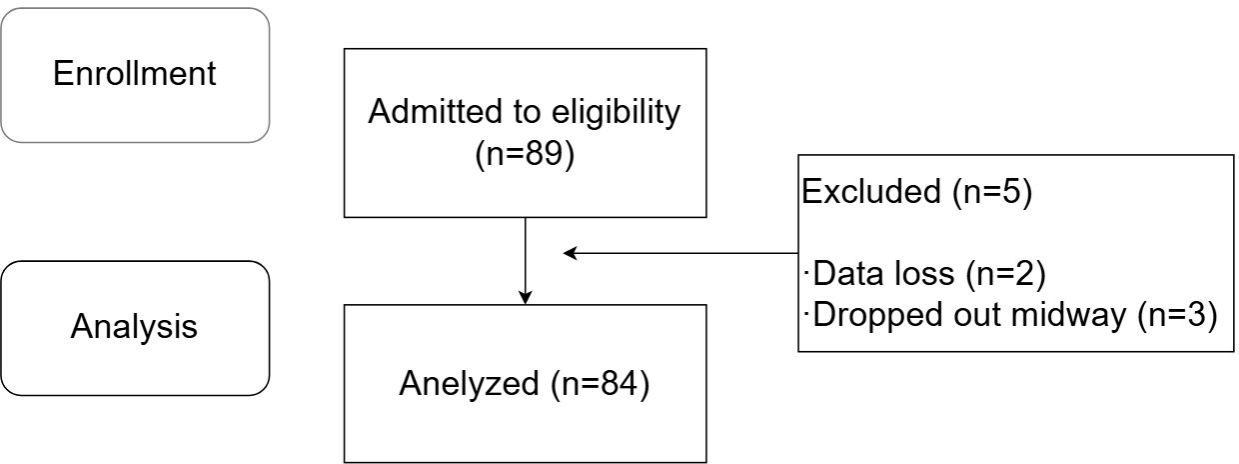
CONSORT diagram. CONSORT indicates Consolidated Standards of Reporting Trial.

A total of 89 patients were enrolled in this study, among whom 3 were excluded due to data loss during collection, and 2 dropped out midway. All participants provided informed consent after being fully informed about the details of the experiment. Ultimately, 84 patients completed the study, with 70 patients in the successful ultrasound-guided ISBPB group and 14 patients in the failed group. The specific experimental process is shown in Figure 2.

The general characteristics of patients in the successful and failed ultrasound-guided ISBPB groups, including age, gender, height, weight, ASA classification, BMI, MAP, and surgical site (left/right), are summarized in Table 1. There was no significant difference between the two groups.

**Table 1:** Baseline characteristics.

Variable	Successful group (*n* = 70)	Failure group (*n* = 14)	*p*-value
Age, y	56.69 ± 6.22	54.00 ± 6.01	0.142
Sex (female/male), *n* (%)	31/39	7/7	0.695
BMI (kg/m^2^)	22.39 ± 1.45	22.52 ± 1.15	0.762
ASA physical status, *n* (%)			
I	29	5	0.691
II	41	9	
MAP (mm Hg)	93.79 ± 9.04	90.93 ± 9.66	0.289
Surgical location			
Left	27	8	0.198
Right	43	6	

Data are presented as median (IQR) and frequency (%).

ASA, American Society of Anesthesiologists; BMI, body mass index; MAP, Mean Arterial Pressure.

**Figure 3:**
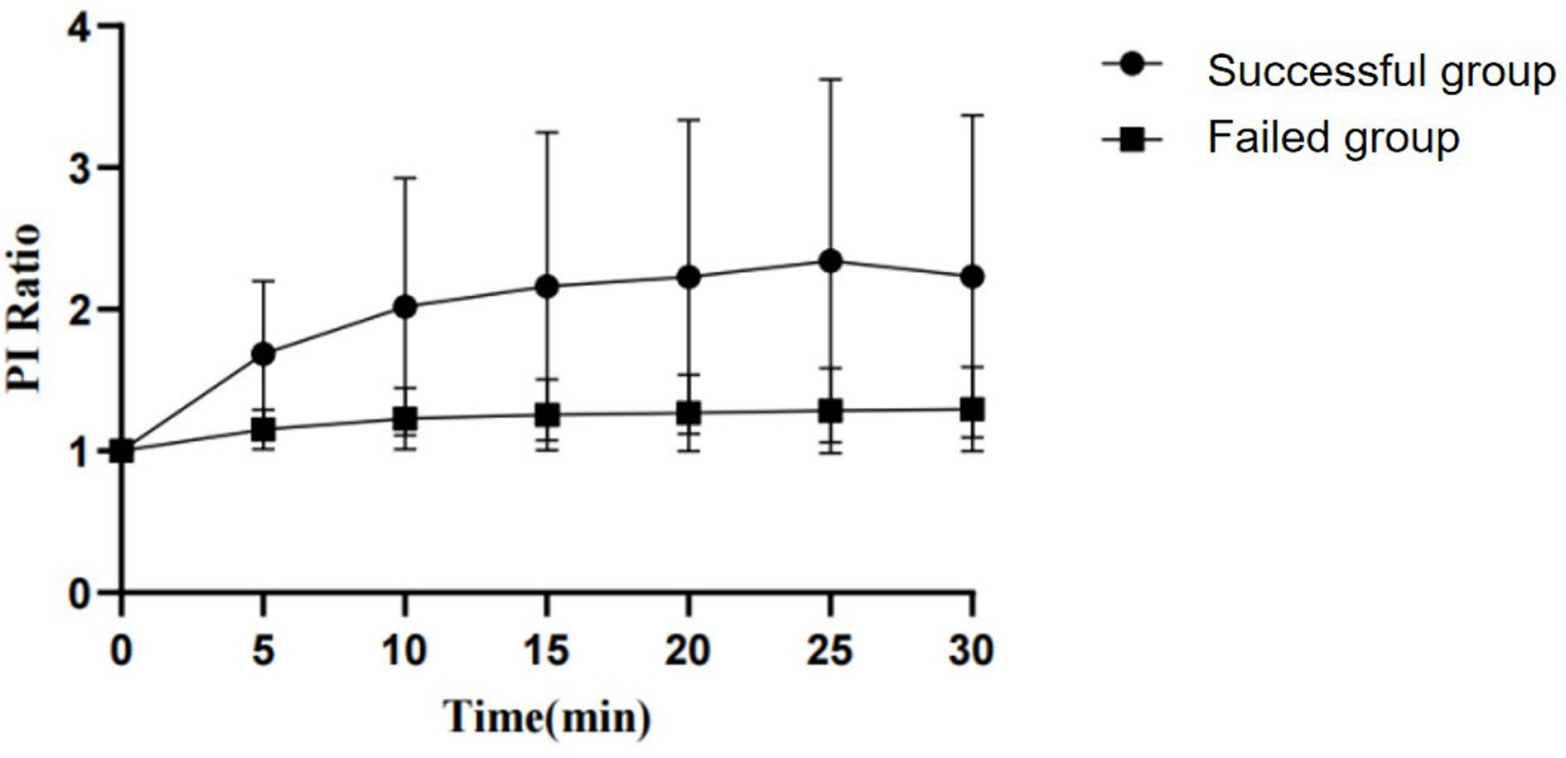
The change in PI ratio over the observation period between the successful and failed groups of ultrasound-guided ISBPB.

**Figure 4:**
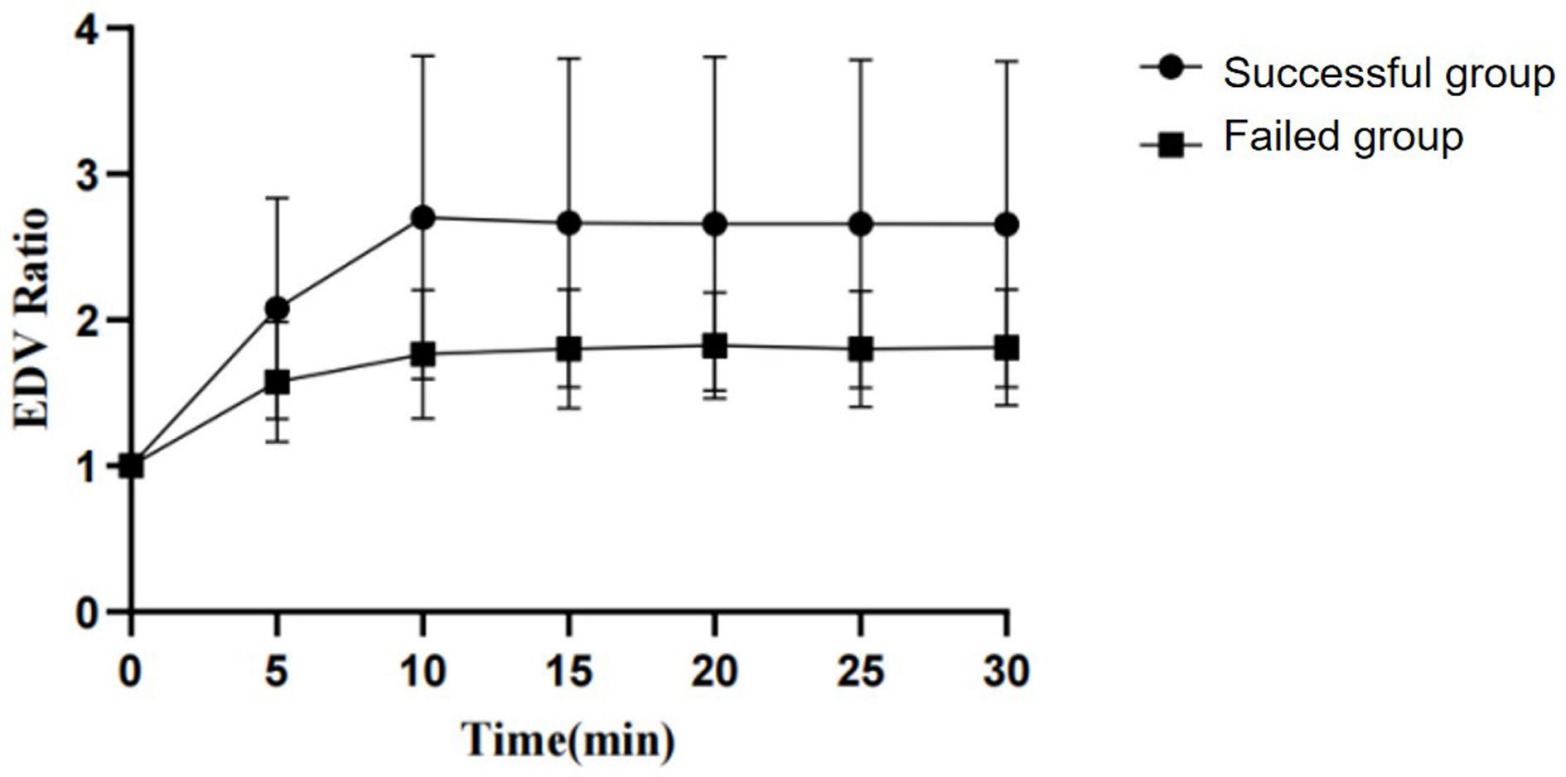
The change in EDV ratio over the observation period between the successful and failed groups of ultrasound-guided ISBPB.

By observing the PI and EDV values at different time points and calculating their ratios to the baseline to convert them into PI ratio and EDV ratio, the results for PI ratio and EDV ratio in the successful ISBPB block group under ultrasound guidance are shown in Table 2. At the 5-min observation point, the PI ratio and EDV ratio significantly increased compared to the baseline point, with a statistically significant difference (*p* < 0.05). Similarly, at other observation points, including 10, 15, 20, 25, and 30 min, the PI ratio and EDV ratio also showed significant increases compared to the baseline point, with statistically significant differences (*p* < 0.05). The trends in PI ratio and EDV ratio over time are illustrated in Figures 3, 4.

**Table 2:** PI ratio and EDV ratio in the successful group.

Variable	PI ratio	EDV ratio
5 min	1.69 ± 0.51*	2.08 ± 0.76*
10 min	2.02 ± 0.91*	2.70 ± 1.11*
15 min	2.16 ± 1.09*	2.66 ± 1.13*
20 min	2.23 ± 1.11*	2.66 ± 1.14*
25 min	2.34 ± 1.28*	2.66 ± 1.12*
30 min	2.23 ± 1.14*	2.66 ± 1.12*

*p < 0.05 indicates a statistical difference compared with baseline.

**Figure 5:**
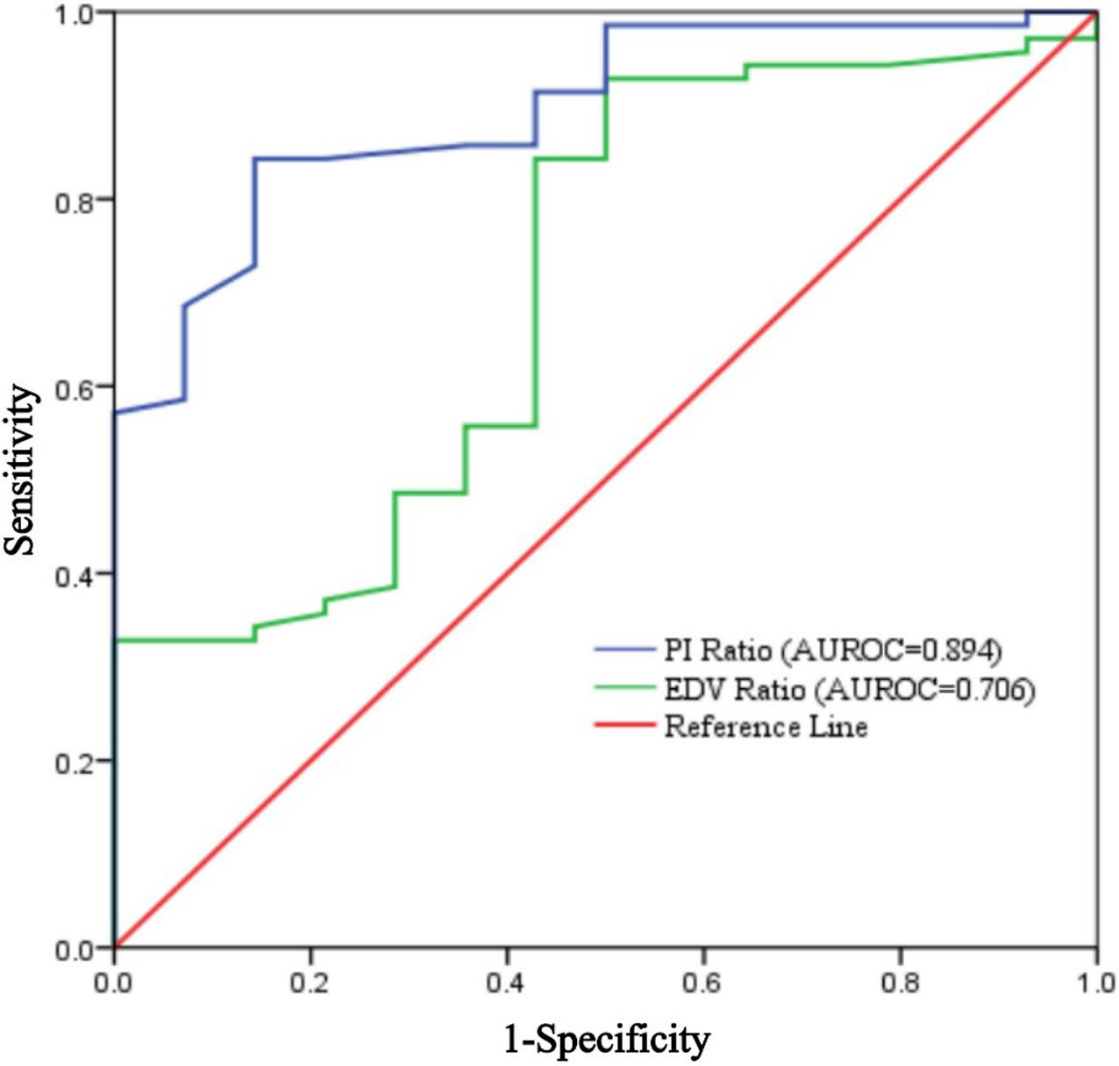
ROC curve of PI ratio and EDV ratio at 5 min.

**Figure 6:**
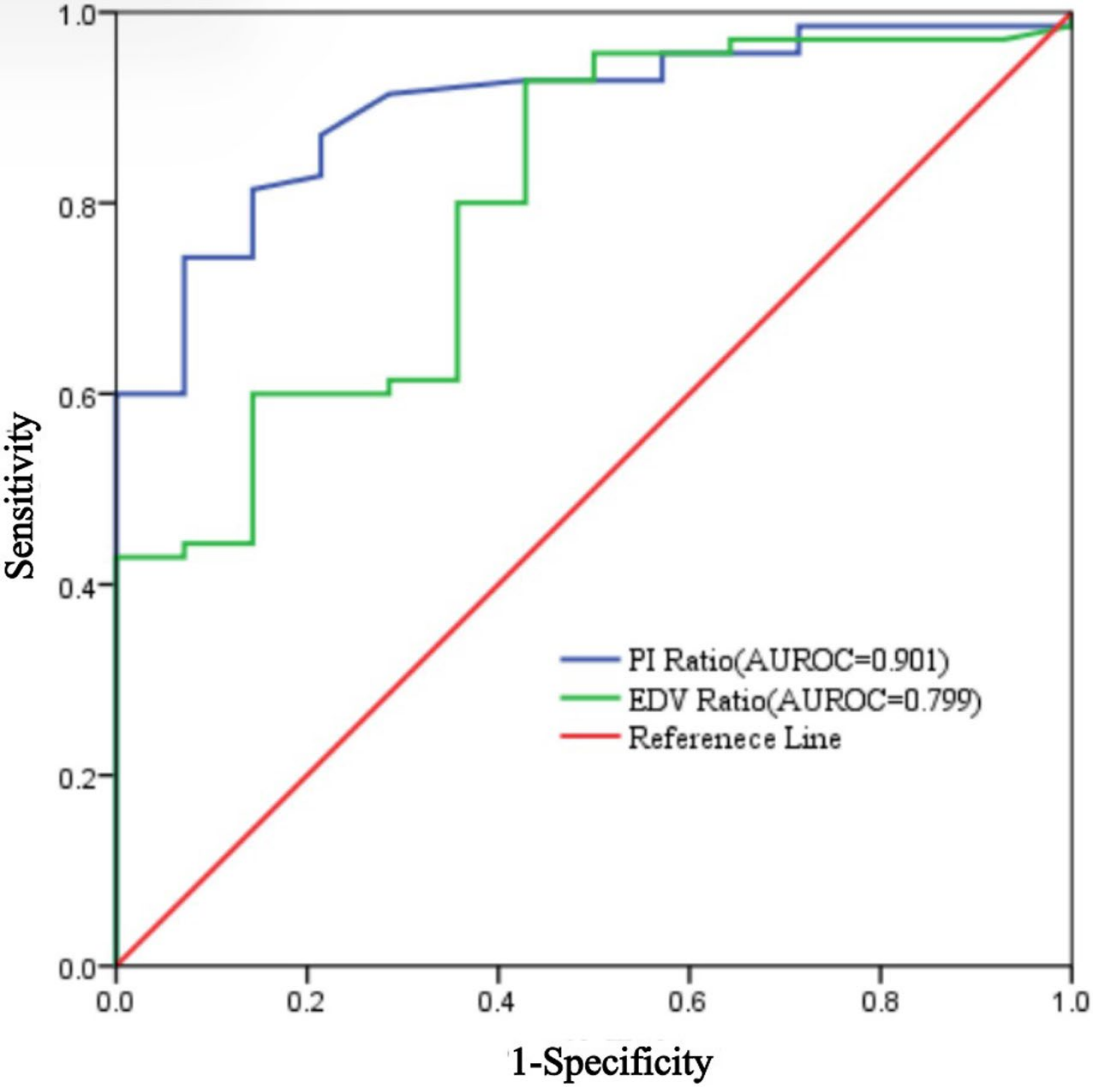
ROC curve of PI ratio and EDV ratio at 10 min.

Using the achievement of sensory loss in at least three skin regions innervated by target nerves within 30 min after ultrasound-guided ISBPB as the criterion for a successful block, this study plotted ROC curves for PI ratio and EDV ratio at 5 and 10 min. The results showed that at 5 min, the threshold for PI ratio to predict the effect of ultrasound-guided ISBPB was 1.22, with a sensitivity of 84.3%, specificity of 85.7%, and an area under the curve (AUC) of 0.894 (95% CI: 0.816–0.972). At 10 min, the threshold for PI ratio was 1.4, with a sensitivity of 74.3%, specificity of 92.9%, and an AUC of 0.901 (95% CI: 0.828–0.974). For EDV ratio, at 5 min, the threshold was 1.32, with a sensitivity of 92.9%, specificity of 50%, and an AUC of 0.706 (95% CI: 0.553–0.860). At 10 min, the threshold was 1.54, with a sensitivity of 92.9%, specificity of 57.1%, and an AUC of 0.799 (95% CI: 0.678–0.921). Details are provided in Table 3 and Figures 5, 6.

**Table 3:** Results of the ROC curve analysis for PI ratios and 5 min and 10 min EDV ratios.

Variable	Time	AUROC	95% CI	Sensitivity	Specificity
PI ratio	5 min	0.894	0.816–0.972	0.843	0.857
	10 min	0.901	0.828–0.974	0.743	0.929
EDV ratio	5 min	0.706	0.553–0.860	0.929	0.500
	10 min	0.799	0.678–0.921	0.929	0.571

CI, confidence interval; ROC, receiver operating characteristic curve.

The Delong test results showed that at 5 min, the AUROC of the PI ratio was greater than that of the EDV ratio, with a statistically significant difference (*p* < 0.05), indicating that the PI ratio had a higher ability to assess the effectiveness of ultrasound-guided ISBPB compared to the EDV ratio at 5 min. Furthermore, there was no statistically significant difference (*p* > 0.05) between the AUROC of the PI ratio at 5 min and at 10 min. Details are provided in Table 4.

**Table 4:** PI ratio and EDV ratio at different times AUROC pairwise contrast results.

Variable	Variable	AU ROC difference	Standard deviation	95% CI	*p*-value
PI ratio (5 min)	EDV ratio (5 min)	0.1878	0.0889	0.014–0.362	0.0346
	PI ratio (10 min)	0.0071	0.0348	−0.061 to 0.075	0.8372

CI, confidence interval; ROC, receiver operating characteristic curve.

## 4 Discussion

In this study, 5 min post-ISBPB, the AUROC for predicting block success was 0.894 (95% CI: 0.816–0.972) using the PI ratio, and 0.706 (95% CI: 0.553–0.860) using EDV. At 5 and 10 min, the AUROC for the PI ratio was 0.901 (95% CI: 0.828–0.974), and for EDV, it was 0.799 (95% CI: 0.678–0.921).

The findings showed that the PI ratio and EDV significantly increased in the success group at 5 and 10 min after ISBPB, but this rise leveled off after 10 min. These changes suggest that PI and EDV ratios are key indicators of nerve block effectiveness, particularly between 5 and 10 min post-procedure. Regional nerve blocks inhibit sensory, motor, and sympathetic nerves, with sympathetic nerves affected first (8, 9). Sympathetic vasoconstrictor fibers, mainly in small arteries and arterioles, are singularly innervated. Blocking these nerves reduces norepinephrine release, relaxing smooth muscle, decreasing peripheral resistance, and increasing blood flow. The pulse oximeter uses photoplethysmography to measure the PI, which is the ratio of pulsatile signals from small arteries to non-pulsatile signals like venous blood and muscle (10). This indicates local blood flow. After a successful block, increased blood flow enhances light absorption in pulsatile tissue, significantly raising the PI ratio within 5 min. Sebastiani et al. conducted an analysis comparing the PI between the blocked and non-blocked sides within the successful group of ISBPB and observed a significant increase in the PI difference at the five-minute mark (11). This finding suggests that the PI at 5 min post-block can serve as a reliable indicator for assessing the efficacy of ISBPB.

The EDV of the brachial artery serves as an indicator of hemodynamic alterations. Following an ISBPB, the suppression of sympathetic activity results in the relaxation of smooth muscle in small arteries and arterioles, thereby causing vasodilation, a reduction in peripheral resistance, and an increase in the EDV of the brachial artery (12). In their study, Li et al. employed spectral Doppler ultrasound to examine the hemodynamic changes in the ipsilateral brachial artery subsequent to an axillary brachial plexus block, identifying the alteration in EDV as the most pronounced among the local hemodynamic parameters (13).

In the failure group, the PI ratio showed minimal change post-nerve block compared to baseline, while the EDV ratio varied. This could be due to the different vessels assessed by EDV and PI, as brachial plexus blockade affects local hemodynamics differently across vessels (14). Variations in vessel diameters may cause different dilation levels in response to nerve blockade. Despite partial effects in the failure group, the EDV ratio difference between success and failure groups remained statistically significant throughout the study.

The PI and EDV ratios showed significant changes at 5 and 10 min post-block, but these changes leveled off after 10 min. Extending the observation period did not improve predictive value, so we focused on 5 and 10 min for analysis. ROC curves indicated that the PI ratio at 5 min had better predictive capability. The Delong test showed no significant difference in predictive performance between 5 and 10 min for the PI ratio. Thus, the 5-min PI ratio effectively indicates block efficacy, offering higher accuracy and earlier assessment, making it a reliable indicator for evaluating ISBPB.

PI is mainly affected by stroke volume and peripheral vascular tone, with systemic or local factors altering these parameters (15). Evidence indicates that PI is more sensitive and specific than traditional subjective evaluation methods (7). It not only indicates the success of regional nerve blocks but also reflects the speed of block onset through its early increase, making it useful for assessing nerve block efficacy in both conscious and sedated patients. A study by Xu et al. found that ketamine sedation does not affect the accuracy of PI in assessing anesthesia effectiveness (16). PI offers earlier and more precise evaluations of caudal anesthesia than HR, MAP, or cremaster reflex indicators. It also allows for quicker assessment of ISBPB effectiveness compared to traditional methods like cold sensation, pinprick, and motor function tests. Recognizing the significant variability in baseline PI values is important. Studies on healthy, awake volunteers showed skewed finger PI values, with distributions of 2.2 ± 2.0% and 3.5 ± 2.4% (17, 18). Thus, using absolute PI values to assess regional nerve block efficacy is unreliable. The PI ratio, which divides PI by its baseline, reduces individual variability and measurement equipment impact. This ratio captures the relative changes following a regional nerve block (19). Abdelnasser et al. compared the PI and PI ratio between the blocked and non-blocked arms within the same patient, identifying a threshold of 1.4 as predictive of the efficacy of a clavicular brachial plexus block at 5 min (20). Kim’s study identified a threshold of 1.6 with epinephrine (21). These studies highlight the PI ratio as a reliable measure for assessing regional nerve block effectiveness. In practice, nerve block success is usually evaluated on the blocked limb. Our study focused on this and found that the PI ratio significantly increased in successful blocks at 5 min, with a cutoff value of 1.22.

The EDV ratio is less accurate than the PI ratio, likely due to factors like probe pressure during Doppler ultrasound or heat loss affecting vascular tone. On the other hand, PI is measured using a pulse oximeter with a relatively stable probe position, and environmental factors such as light and temperature interference can be minimized by shielding the sensor, maintaining appropriate temperature, and wrapping the sensor to reduce disturbances. This enhances the PI ratio’s accuracy and reliability, offering non-invasive, user-friendly, and continuous monitoring benefits, which are highly valuable in clinical settings.

This study has several limitations: pinprick tests were only done at 30 min, missing other time points for pain assessment; it focused solely on awake patients, excluding those sedated or anesthetized; and it used a ropivacaine-lidocaine mix, needing further research on different anesthetic types and concentrations.

In summary, the PI ratio is a dependable measure for evaluating ISBPB efficacy, offering a precise and early assessment at 5 min.

## Data Availability

The raw data supporting the conclusions of this article will be made available by the authors without undue reservation.

## Footnotes

^1^https://www.chictr.org.cn/showproj.html?proj=159766; 20 December 2022.
